# Phenylpropanoid metabolites from grape leaves contribute to strong defense roles against downy mildew based on physiological and transcriptomic analyses

**DOI:** 10.3389/fmicb.2026.1805591

**Published:** 2026-03-17

**Authors:** Zhili Xun, Haijin Qin, Feng Li, Zhaoqian Yao, Yifan Xu, Min Wang, Liping Huang, Yue Zhu, Xueqing Geng, Qifeng Zhao

**Affiliations:** 1 Institute of Pomology, Shanxi Agricultural University/Shanxi Key Laboratory of Germplasm Improvement and Utilization in Pomology, Taiyuan, Shanxi, China; 2 School of Agriculture and Biology, Shanghai Jiao Tong University, Shanghai, China

**Keywords:** differentially expressed genes, grape downy mildew, phenylpropanoid metabolites, *Plasmopara viticola*, RNA-seq

## Abstract

Downy mildew caused by *Plasmopara viticola* seriously affects global grape production. In this study, the cultivars of Moldova and Red Globe were inoculated with *Plasmopara viticola* separately. The results showed that the inoculated sites on Red Globe leaves were densely covered with white cotton-like mycelial mats, whereas sparse white mycelia were observed on Moldova leaves. Disease index analysis of Moldova and Red Globe after inoculation with the pathogen showed that Red Globe had a much higher disease index than Moldova, indicating that Red Globe was a highly resistant cultivar, while Moldova was a susceptible cultivar. Furthermore, it was discovered that Moldova had significantly higher contents of lignin, total phenols, and flavonoids than Red Globe. Moreover, RNA-seq technology was used to analyze transcriptomic changes in Red Globe and Moldova after inoculation at 1 dpi and 5 dpi separately. Combined with GO and KEGG databases to annotate differentially expressed genes (DEGs), the DEGs related to the plant hormone signal transduction pathway (*VvPR1, VvJAR1, VvSUC11*, etc.), phenylpropanoid metabolism (*VvCAD, VvCCoAOMT*, etc.), flavonoid biosynthesis pathways (*VvCHS, VvF3H, VvDFR*, etc.), and the stilbenoid, diarylheptanoid, and gingerol biosynthesis pathway (*VvROMT, VvSTSs*) were further analyzed. Furthermore, the results showed that Moldova activated more disease-resistant genes in the phenylpropanoid metabolic pathway, and the expression levels of genes related to disease-resistant pathways were significantly higher than those in Red Globe. This study lays a foundation for screening candidate target genes for downy mildew-resistant grape breeding and for analyzing the interaction mechanism between the pathogen and grapes.

## Introduction

*Vitis vinifera* L. is a perennial woody vine belonging to the genus *Vitis* (family *Vitaceae*), with significant economic and ecological value worldwide. However, grape production is severely threatened by downy mildew, a devastating disease caused by the obligate biotrophic oomycete pathogen *Plasmopara viticola*, particularly under favorable humid conditions (Chen et al., 2019). *P. viticola* primarily infects young and vulnerable tissues such as grape leaves, fruit clusters, and shoots, leading to extensive defoliation, fruit rot, and ultimately substantial yield losses and quality deterioration (Zhang et al., 2023; Li et al., 2020). Moreover, the long-term and excessive use of fungicides has driven the gradual evolution of *P. viticola* resistance, resulting in drastically reduced fungicidal efficacy and posing serious challenges to sustainable grape production (Clippinger et al., 2024). Thus, it is necessary to investigate the molecular mechanisms underlying *P. viticola* resistance and identify key resistance-related genes, as this is an urgent and crucial strategy for developing environmentally friendly downy mildew management approaches, which is of great practical significance for safeguarding the grape industry.

The phenylpropanoid metabolic pathway is one of the most diverse and evolutionarily conserved secondary metabolic pathways in plants, serving as a core hub for plant defense against biotic stresses. The phenylpropanoid metabolites, including lignin, flavonoids, and phenolic acids, participate not only in plant structural support, ultraviolet protection, and pigment formation but also in mediating defense responses against pathogens and herbivores (Vogt, 2010; Stout and Chapple, 2004). With advances in multi-omics technologies, the phenylpropanoid metabolic pathway has been extensively investigated in model plants such as *Arabidopsis thaliana*, cucumber, rice, and hulless barley (Stout and Chapple, 2004; Cheynier et al., 2013; Mansfeld et al., 2017). These studies have demonstrated that the phenylpropanoid metabolic pathway is a highly dynamic and regulatable system that can precisely coordinate plant responses to environmental challenges. Key enzymes in this pathway, including phenylalanine ammonia-lyase (PAL), cinnamate 4-hydroxylase (C4H), and 4-coumarate: coenzyme A ligase (4CL), exhibit tissue-specific and stress-responsive expression patterns that are tightly linked to stress resistance (Rahman et al., 2022). It has been previously reported that fulvic acid can protect table grapes against *Plasmopara viticola* infection by activating the phenylpropanoid metabolic pathway, inducing the accumulation of phenolic compounds and flavonoids, enhancing the activity of key enzymes, and upregulating the transcription of related biosynthetic genes, with no direct antifungal activity (Xu et al., 2024). *ZmCCoAOMT2*, which encodes the caffeoyl-CoA O-methyltransferase in maize, has been identified as a quantitative trait locus (QTL) conferring broad-spectrum resistance to southern leaf blight, gray leaf spot, and northern leaf blight (Yang et al., 2017). The accumulation of antifungal metabolites, including ferulic acid and quercetin, was identified following infection of *Lilium brownii var. viridulum* with *Fusarium oxysporum* (Deng et al., 2024). The natural resistance of *Humulus lupulus* L. to downy mildew (*Pseudoperonospora humuli*) is associated with the prophylactic synthesis of phenylpropanoids, and exogenous application of these compounds can reduce the extent of infection in susceptible genotypes (Feiner et al., 2021). As core phytoalexins in *Vitaceae*, stilbenes and their synthetic pathway activation are key resistance traits in grapes against downy mildew (*Plasmopara viticola*), with an antagonistic expression pattern of stilbene synthase and chalcone synthase genes between resistant and susceptible genotypes (Ciaffi et al., 2020). Furthermore, exogenous methyl jasmonate (MeJA) treatment of susceptible grape leaves induced the accumulation of stilbene synthase, thereby enhancing disease resistance (Belhadj et al., 2006), and the stilbene content in infected grape leaves could distinguish susceptible and resistant cultivars (Schnee et al., 2008). Collectively, these studies establish a theoretical foundation for understanding plant disease resistance, highlighting the roles of secondary metabolic pathways and phenylpropanoid-related gene expression in pathogen defense. In particular, activation of the phenylpropanoid pathway and the resulting metabolites are key regulators of disease resistance.

However, our understanding of the comprehensive regulatory network of the phenylpropanoid metabolic pathway in grape response to *P. viticola* remains limited. Specifically, the dynamic changes of phenylpropanoid pathway-related metabolites and the key candidate genes mediating the resistance differences between resistant and susceptible grape cultivars under *P. viticola* infection have not been systematically elucidated, which further hinders the development of targeted resistance breeding strategies for grape downy mildew.

In this study, we analyzed the dynamic changes in grape phenylpropanoid pathway-related metabolites in both resistant (Moldova) and susceptible (Red Globe) materials after 1 day post-infection (dpi) and 5 dpi post-inoculation by the obligate biotroph *P. viticola*. We screened key candidate genes associated with the phenylpropanoid metabolic pathway under *P. viticola* invasion. Furthermore, we used RNA sequencing (RNA-seq) to systematically investigate the transcriptional responses of grapevines infected with *P. viticola*, to identify differentially expressed genes (DEGs) following *P. viticola* invasion between grape resistant and susceptible genotypes. Gene Ontology (GO) terms and Kyoto Encyclopedia of Genes and Genomes (KEGG) databases have been used to annotate functions of differentially expressed genes (DEGs) in Moldova and Red Globe. We focused on the analysis of the high enrichment of “metabolic process” and “catalytic activity,” which indicates that plants initiate extensive metabolic reprogramming in response to pathogen stress, particularly the biosynthesis of defense-related secondary metabolites. Together, these findings will not only help us understand the molecular mechanisms underlying phenylpropanoid-mediated *P. viticola* resistance but also provide valuable gene resources and a theoretical basis for future breeding of *P. viticola*-resistant grape cultivars, thereby contributing to the sustainable development of the global grape industry.

## Materials and methods

### Plant materials and stain

In April 2025, Moldova and Red Globe were collected from the Taigu Grape Nursery of National Germplasm Resources (located at 37°23′N, 112°32′E, at an altitude of 830 meters, with an annual average temperature of 10.6 °C, a frost-free period of 160–180 days, and an annual precipitation of 400–600 millimeters). This base adopts a north-south planting method, with a row spacing of 2.3 meters × 1.4 meters, and the plants are supported by Y-shaped supports. All other cultivation, pest, and disease control measures are carried out using conventional methods. The collected samples are the fully expanded healthy leaves from the 4th to 6th nodes at the top of each grape variety. Red Globe is an Eurasian variety, diploid; the parents are Emperor × S45-48, a multi-parent hybrid seedling. It has long conical clusters, round or oval berries, and deep red to purplish-red skin. Moldova is a European-American hybrid variety, diploid; the parents are GuzaliKala × SV12375. It has conical clusters, short elliptical berries, and blue-black skin. The *Plasmopara viticola* strain (GX5-12) was kindly provided by Dr. Jiang Lu's lab from Shanghai Jiao Tong University. This strain was isolated from naturally infected grape leaves in Guangxi Province and maintained on susceptible Red Globe grape leaves under 25 ± 2 °C and 95% relative humidity. The sporangial suspension was prepared at a concentration of 1 × 10^5^ sporangia mL^−1^ for inoculation.

### Sample preparation

The leaves from the 4th to 6th nodal positions of the shoot apex of each plant were collected, rinsed with water, punched into 1.5 cm diameter leaf disks, and then placed abaxial side up, lined with two layers of moist sterilized filter paper in petri dishes. Leaves were inoculated with *P. viticola* sporangial suspension at a concentration of 1 × 10^5^ sporangia·mL^−1^. All prepared dishes were incubated in a growth chamber at 22 °C with a 16 h light/8 h dark photoperiod. Non-inoculated leaf disks were used as the control. Each treatment was four biological replicates, with 50 leaf disks per replicate. Collected leave samples from 0 h, 1 dpi, and 5 dpi were immediately frozen in liquid nitrogen and stored at −80 °C for subsequent experiments.

The disease index of each cultivar was calculated based on the grape downy mildew leaf grading standard (Supplementary Table S1) using the following formula:


$$\begin{array}{l} \text{Disease index = }[\Sigma (\text{Number of leaf disks at each grade}\\ \;\times \text{Corresponding grade value})\text{/}\\ \;(\text{Total number of leaf disks}\times \text{Maximum grade}\\ \text{ value})]\times \;\text{100} \end{array}$$

### Chlorogenic acid content determination

The chlorogenic acid content of inoculated leaves was measured using a method with minor modifications (Jiang et al., 2021). Briefly, the dried leaves were ground into a fine powder, and 0.5 g of the powder was transferred to a flask containing 70% (v/v) ethanol. The mixture was extracted in a water bath at 55 °C for 2.5 h, centrifuged at 8,000 g/min for 10 min, and the supernatant was collected as the crude chlorogenic acid extract. 0.2 g of activated carbon was added, and the mixture was vigorously shaken for 15 min, then allowed to stand for 5 min, and filtered through a 0.45 μm organic phase filter membrane to obtain a clear sample solution. A series of chlorogenic acid standard solutions with gradient concentrations was prepared to plot a standard curve. With 70% ethanol as the blank control, the absorbance of the sample solution was measured at 327 nm by a spectrophotometer. Chlorogenic acid concentration in the sample solution was calculated based on the standard curve, and the content in grape leaves (mg/g dry weight, DW) was converted by accounting for sample weight, extraction volume, and dilution factor.

### Lignin, total phenol, and flavonoid content determination

For lignin assay, 50 mg of precipitated sample was dried, centrifuged, washed with 95% ethanol, and incubated with 1 mL of 25% bromoacetyl bromide-acetic acid at 70 °C for 30 min. The reaction was terminated by adding 1 mL of 2 M NaOH, followed by the addition of 2 mL of acetic acid and 3.75 M hydroxylamine hydrochloride, and centrifugation (8,000 g/min, 4 °C, 10 min). 0.5 mL of the supernatant was pipetted, diluted to 15 mL with acetic acid, and the absorbance was measured at 280 nm. For total phenol and flavonoid assays: 2 g of tissue samples from the junction of diseased and healthy tissues were homogenized in 20 mL of 1% (v/v) hydrochloric acid-methanol at 4 °C. After extraction, the homogenate was centrifuged in the dark (12,000 g/min, 4 °C, 10 min). The total phenol and flavonoid contents in the supernatant were determined by measuring absorbance at 280 nm and 325 nm, respectively (Zhu et al., 2016).

### Malondialdehyde (MDA) content determination

MDA content was measured using the thiobarbituric acid (TBA) method (Shannon et al., 1966). 0.5 g of frozen leaf powder was mixed with 5 mL of 0.1% trichloroacetic acid (TCA) solution, ground into a homogenate in an ice bath, and centrifuged at 8,000 g/min for 10 min. Two mL of the supernatant was mixed with 2 mL of 0.5% TBA solution (dissolved in 20% TCA), vortexed thoroughly, heated in a boiling water bath for 30 min, and rapidly cooled to room temperature in an ice bath. After cooling, the mixture was centrifuged again (8,000 g/min, 10 min), and the absorbance of the supernatant was measured at 532 nm and 600 nm with a TCA-TBA mixture as the blank control. MDA content (μmol/g FW) was calculated using the formula:


$$\begin{array}{lll} MDA & = & \left[6.45\times \left(A_{532}-A_{600}\right)-0.56\times A_{440}\right]\times Extraction\\ volume/Fresh\;sampleweight \end{array}$$

### Peroxidase (POD) activity assay

For POD activity measurement, 0.5 g of leaves was homogenized in 0.05 mol/L phosphate buffer (pH 6.0, containing 1% polyvinylpyrrolidone, PVP) on an ice bath, then centrifuged at 10,000 g/min for 15 min. The supernatant was used as the crude enzyme extraction. The reaction system (3.0 mL total volume) consisted of 2.5 mL of 0.05 mol/L phosphate buffer (pH 6.0), 0.2 mL of 0.02 mol/L guaiacol, 0.2 mL of 0.01 mol/L H_2_O_2_, and 0.1 mL of crude enzyme extract (added last to initiate the reaction). At 25 °C, the spectrophotometer was zeroed using a blank control, and the absorbance was measured continuously at 470 nm for 3 min with readings recorded every 30 s. One POD activity unit (U) was defined as an increase of 0.01 in absorbance per minute; the total activity over 60 min was calculated, and the specific activity was expressed as U/g FW·h (Shannon et al., 1966).

### Polyphenol oxidase (PPO) activity assay

For POD activity, 0.5 g of leaves was homogenized in 5 mL of 0.1 mol/L phosphate buffer (pH 6.5, containing 1% PVP) on an ice bath, then centrifuged at 12,000 g/min for 20 min; the supernatant was collected as the crude enzyme extraction. The reaction system (3.0 mL total volume) consisted of 2.7 mL of 0.1 mol/L phosphate buffer (pH 6.5), 0.2 mL of 0.05 mol/L catechol (substrate), and 0.1 mL of crude enzyme extract (added last to trigger the reaction). At 25 °C, the spectrophotometer was zeroed with a blank control, and the absorbance change rate was measured continuously at 420 nm for 5 min. One PPO activity unit (U) was defined as an increase of 0.01 in absorbance per minute, and the specific activity was expressed as U/g FW·min (Siddiq and Dolan, 2017).

### RNA extraction, library preparation, and sequencing

Total RNA was extracted from *P. viticola*-inoculated leaf tissues at 0 h, 1 dpi, and 5 dpi using a Plant Total RNA Extraction Kit (Bestjay Biotechnology Co., Ltd., Shanghai, China). RNA quality and integrity meeting strict QC criteria (OD_2_60_/2_80 ≈ 2.0, OD_2_60_/2_30 > 2.0, RIN ≥ 8.0) were used for cDNA library construction. Library preparation was performed following the standard Illumina protocol by Wuhan Metware Biotechnology Co., Ltd. (Wuhan, China). Briefly, mRNA with poly(A) tails was enriched using Oligo (dT) magnetic beads. First-strand cDNA was synthesized with random hexamer primers, followed by the generation of second-strand cDNA. Purified double-stranded cDNA was subjected to end repair, 3′-end adenylation, and sequencing adapter ligation. Finally, cDNA fragments were selected and enriched by PCR amplification to construct the final cDNA library. Library quality was initially quantified with a Qubit fluorometer, and the insert fragment size was measured using a fragment analyzer. Qualified libraries were sequenced on the Illumina NovaSeq 6000 platform with paired-end (PE150) reads (Wuhan, Hubei, China).

### Sequencing data processing

Raw sequencing reads were subjected to quality control and filtering using fastp (v0.23.2) to obtain high-quality clean reads with the following filtering criteria: Raw reads were first filtered by removing sequences containing sequencing adapters. Reads with more than 10% unknown bases (*N*) were then discarded, followed by elimination of reads in which low-quality bases (Q ≤ 20) accounted for more than 50% of the total bases.

Clean reads were aligned to the *Vitis vinifera* reference genome (Ensembl release-52) using HISAT2 (v2.2.1). The number of reads mapped to each gene was counted using featureCounts (v2.0.3), and gene expression levels were quantified as fragments per kilobase of transcript per million mapped reads (FPKM). Differential expression analysis between inoculated and control samples was performed using the DESeq2 (v1.22.1) R package, with differentially expressed genes (DEGs) screened under the criteria of |log_2_Fold Change| ≥ 1 and false discovery rate (FDR) < 0.05. For the identified DEGs, Gene Ontology (GO) functional annotation and enrichment analysis, as well as Kyoto Encyclopedia of Genes and Genomes (KEGG) pathway enrichment analysis, were performed using the clusterProfiler R package (v4.6.0) (Developed and maintained by Guangchuang Yu, Southern Medical University, Guangzhou, China). All bioinformatics analyses were performed by Wuhan Metware Biotechnology Co., Ltd. The RNA-seq data for the Moldova and Red Globe strains inoculated with the *P. viticola* at 0 h, 1 dpi, and 5 dpi are available at the NCBI Gene Expression Omnibus server (accession no: SAMN54461750).

### Validation of RNA-seq results by quantitative real-time reverse transcription polymerase chain reaction (qRT-PCR)

To verify the accuracy and reliability of the RNA-Seq data, ten DEGs were randomly selected for qRT-PCR validation. Total RNA was extracted using a Plant Total RNA Extraction Kit (Bestjay Biotechnology Co., Ltd., Shanghai, China). The RNA quality was evaluated by 1% (w/v) agarose gel electrophoresis, and RNA concentration was determined using a NanoDrop spectrophotometer, whereas first-strand cDNA synthesis was carried out using the Prime Script™ RT and the gDNA Eraser (TaKaRa, Shanghai, China). Quantitative real-time RT-PCR (qRT-PCR) was performed on a Bio-Rad real-time PCR system (CA, USA) using the SYBR Premix Ex Taq II Kit (Ranbode Biotechnology Co., Ltd., Shanghai, China). The 20 μL reaction system consisted of 10 μL of SYBR Premix Ex Taq II, 0.4 μL of each forward and reverse primer (10 μM), 2 μL of cDNA template, and 7.2 μL of sterile ddH_2_O. The program was as follows: pre-denaturation at 95 °C for 30 s, followed by 40 cycles of denaturation at 95 °C for 10 s and annealing/extension at 62 °C for 30 s. *VvActin1* was used as the reference gene. Relative gene expression levels were calculated using the 2^−ΔΔ*Ct*^ method as previously described (Gao and Chen, 2018; Zhang et al., 2021). The primer sequences for the 10 selected genes are shown in Supplementary Table S2. Each treatment used three independent biological replicates.

### Data statistics and analysis

Statistical analyses were performed with SPSS 19.0 software, including one-way analysis of variance (ANOVA) and Duncan's multiple range test for the comparison of means (*P* < 0.05). Data visualization and plot construction were performed using Origin 2021 and R/Performance Analytics software. Metabolomics-related data analysis was performed using MetaboAnalyst 5.0 (Developed by Xia Lab, McGill University, Montreal, Canada) (https://www.metaboanalyst.ca/docs/RTutorial.xhtml). All experiments were conducted with at least three biological replicates, and results were expressed as the mean ± standard deviation (SD).

## Results

### Phenotypic changes in grape leaves following *P. viticola* inoculation

**Figure 1:**
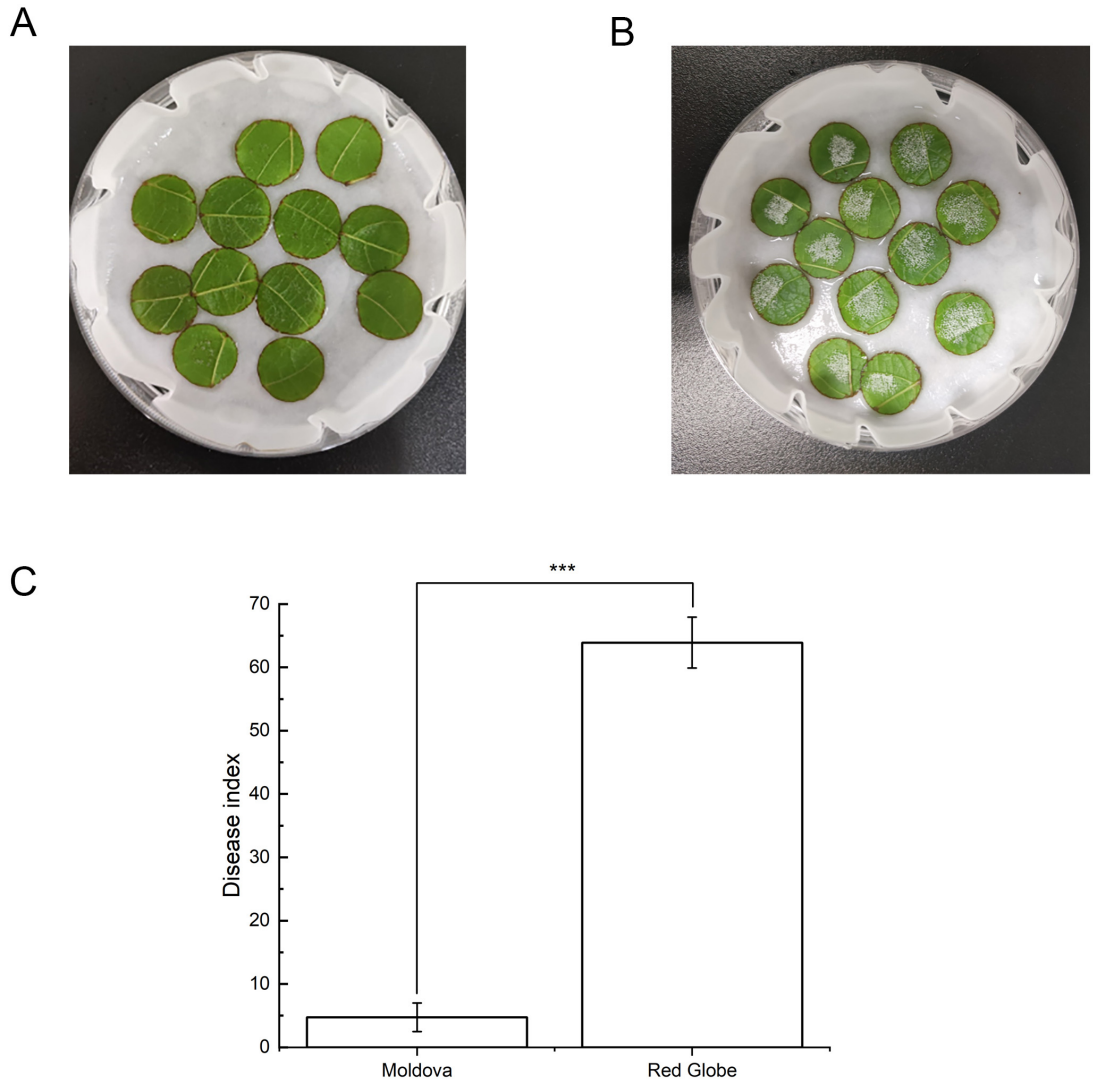
The phenotypic symptoms of leaves infected by *Plasmopara viticola* after 5 days. The picture showed the phenotypic images of 12 representative leaf disks after infection with *Plasmopara viticola* from the grape variety of Moldova **(A)** and Red Globe **(B)**. The disease index of the leaves was recorded 5 days after inoculation with *Plasmopara viticola* according to the grading standard **(C)** (Supplementary Table S1). Data are shown as mean ± SD (*n* = 3). Statistical significance was determined by Student's *t*-test (*p < 0.05, **p < 0.01, ***p < 0.001).

To test the distinct extent of resistance to the pathogen of *P. viticola, two* grape cultivars from Moldova and Red Globe were compared after *P. viticola* infection. Based on our observation, there was a significant difference in the resistance to *P. viticola* between Moldova and Red Globe. At 5 dpi, visual inspection revealed that the inoculated sites on Red Globe leaves were densely covered with white cotton-like mycelial mats, a typical diagnostic feature of downy mildew caused by *P. viticola*. Conversely, only sparse white mycelia were observed on Moldova leaves, and inoculation sites showed no obvious mycelial growth (Figures 1A, B). These results suggested that Moldova has higher resistance than Red Globe after invasion by *P. viticola*.

Furthermore, we conducted a quantitative analysis to detect divergence of the resistance levels between these two cultivars. The results showed that the disease index of Moldova was 4.75, whereas that of Red Globe was 63.90 (Figure 1C). Therefore, Moldova was categorized as a highly resistant (HR) cultivar, with a resistance grade of Grade 1, and Red Globe was classified as a highly susceptible (HS) cultivar with a disease resistance grade of Grade 9 based on the disease resistance classification criteria (Supplementary Table S1). Together, these results indicated that the two selected grape cultivars are suitable for subsequent studies aimed at discovering the molecular mechanism of grape resistance to downy mildew.

### Changes in secondary metabolite contents of grape leaves under *P. viticola* inoculation

**Figure 2:**
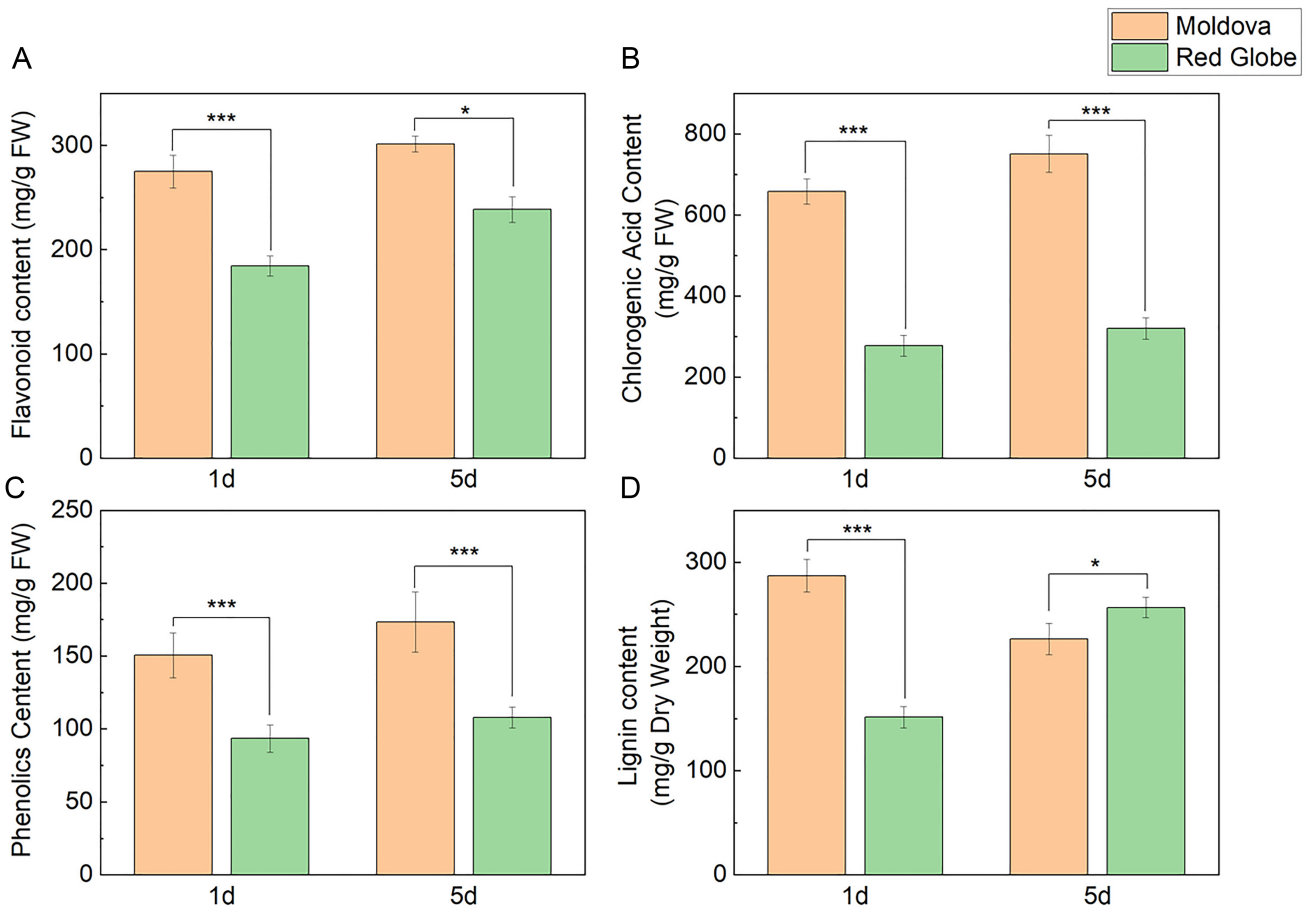
The content of secondary metabolites, including flavonoids, chlorogenic acid, total phenols, and lignin, in grape leaves. Showing are the contents of flavonoids **(A)**, chlorogenic acid **(B)**, total phenols **(C)**, and lignin **(D)** in grape leaves after 1 day and 5 days of *Plasmopara viticola* treatment with Moldova and Red Globe. Data are shown as mean ± SD (*n* = 3). Statistical significance was determined by Student's *t*-test (*p < 0.05, **p < 0.01, ***p < 0.001).

Secondary metabolites, including flavonoids, chlorogenic acid, total phenols, and lignin, all of which are phenylpropanoid metabolites, play important roles in plant and microbe interactions. In this study, we want to determine if these phenylpropanoid metabolites also function as resistance substances during *P. viticola* infection of grape leaves. Therefore, we detected the relative contents of a series of these metabolites. The results showed the resistant cultivar Moldova maintained higher metabolite levels of flavonoid, chlorogenic acid, and total phenols than Red Globe at both 1 and 5 dpi (Figures 2A–C). Interestingly, regarding lignin content, the Red Globe cultivar reached a slightly higher level than that of Moldova only at 5 dpi (Figure 2D). These results indicated that all of these phenylpropanoid metabolites play defense roles after *P. viticola* infection, especially in the resistant cultivar, which initiates a rapid early-stage defense response in grape leaves.

### Antioxidant enzyme activities and Malondialdehyde (MDA) content of grape leaves under *P. viticola* stress

**Figure 3:**
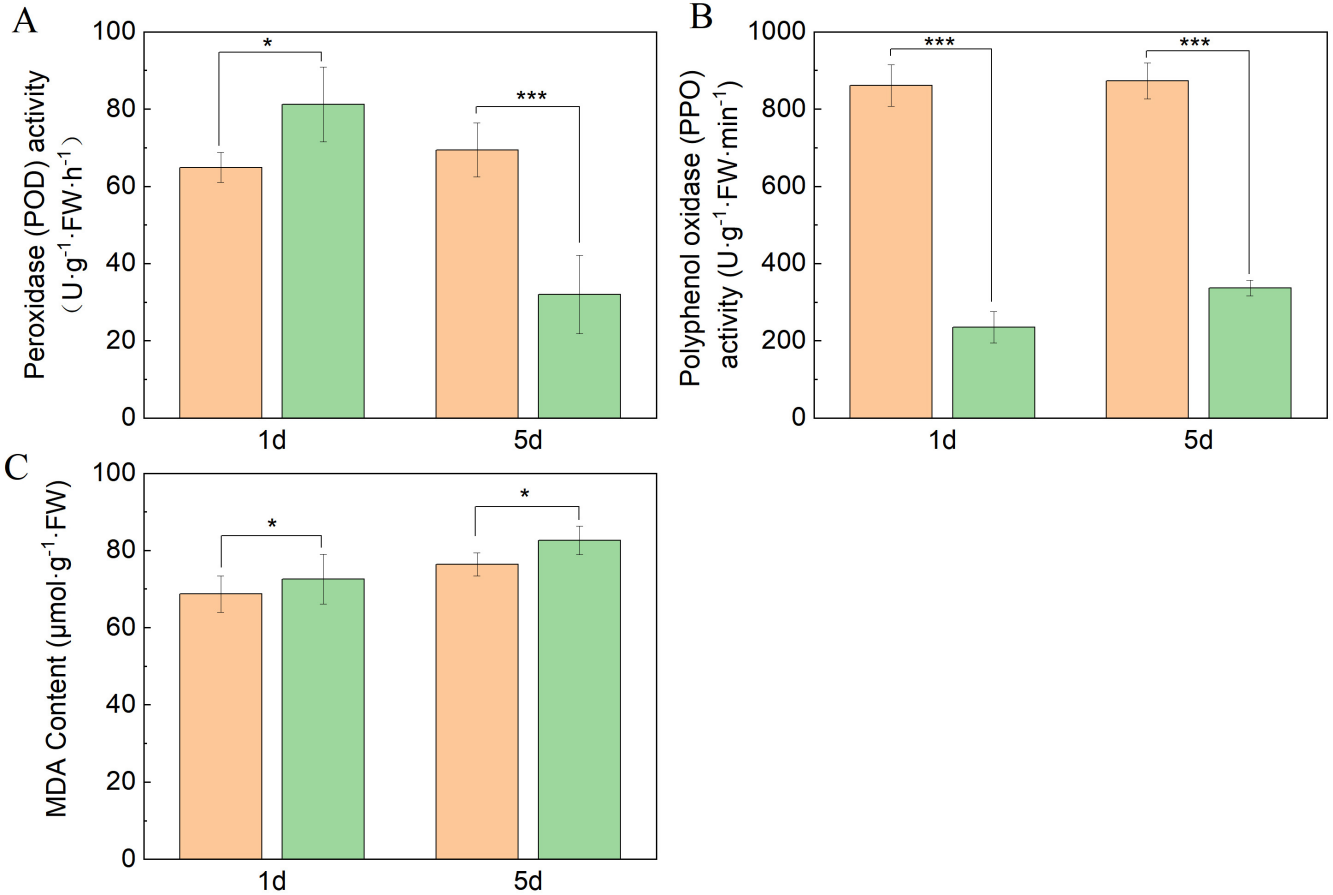
The activity of antioxidant enzymes (PPO, POD) and MDA content in grape leaves after 1 day and 5 days of *Plasmopara viticola* treatment. The activity of PPO **(A)**, POD **(B)**, and MDA **(C)** was determined. The X-axis represents the grape leaf samples at different periods (1 dpi and 5 dpi), and the Y-axis represents the activity or contents of different metabolites. Data are shown as mean ± SD (*n* = 3). Statistical significance was determined by Student's *t*-test (*p < 0.05, **p < 0.01, ***p < 0.001).

Polyphenol oxidase (PPO) and peroxidase (POD) are antioxidant enzymes in plants to scavenge reactive oxygen species (ROS) and mitigate pathogen-induced oxidative damage (Mittler, 2002). We measured these two antioxidant activities. Our results discovered that PPO and POD activities exhibited consistent patterns at 5 dpi after *P. viticola* invasion, the resistant cultivar Moldova maintained significantly higher enzyme activities than the susceptible Red Globe (Figures 3A, B), but for POD activities at 1 dpi, the cultivar Red Globe reached a slightly higher level than Moldova. These results indicated the resistant cultivar had stronger antioxidant enzyme activities to scavenge ROS as the invasion process lasted. As for Malondialdehyde (MDA), a characteristic product of membrane lipid peroxidation, it can reflect the degree of stress-induced damage in plants (Hodges et al., 1999). In this result, Moldova retained lower MDA content than Red Globe at both 1 dpi and 5 dpi (Figure 3C), indicating a milder membrane damage in the resistant cultivar than in the susceptible cultivar.

### Overview of the transcriptome data

**Figure 4:**
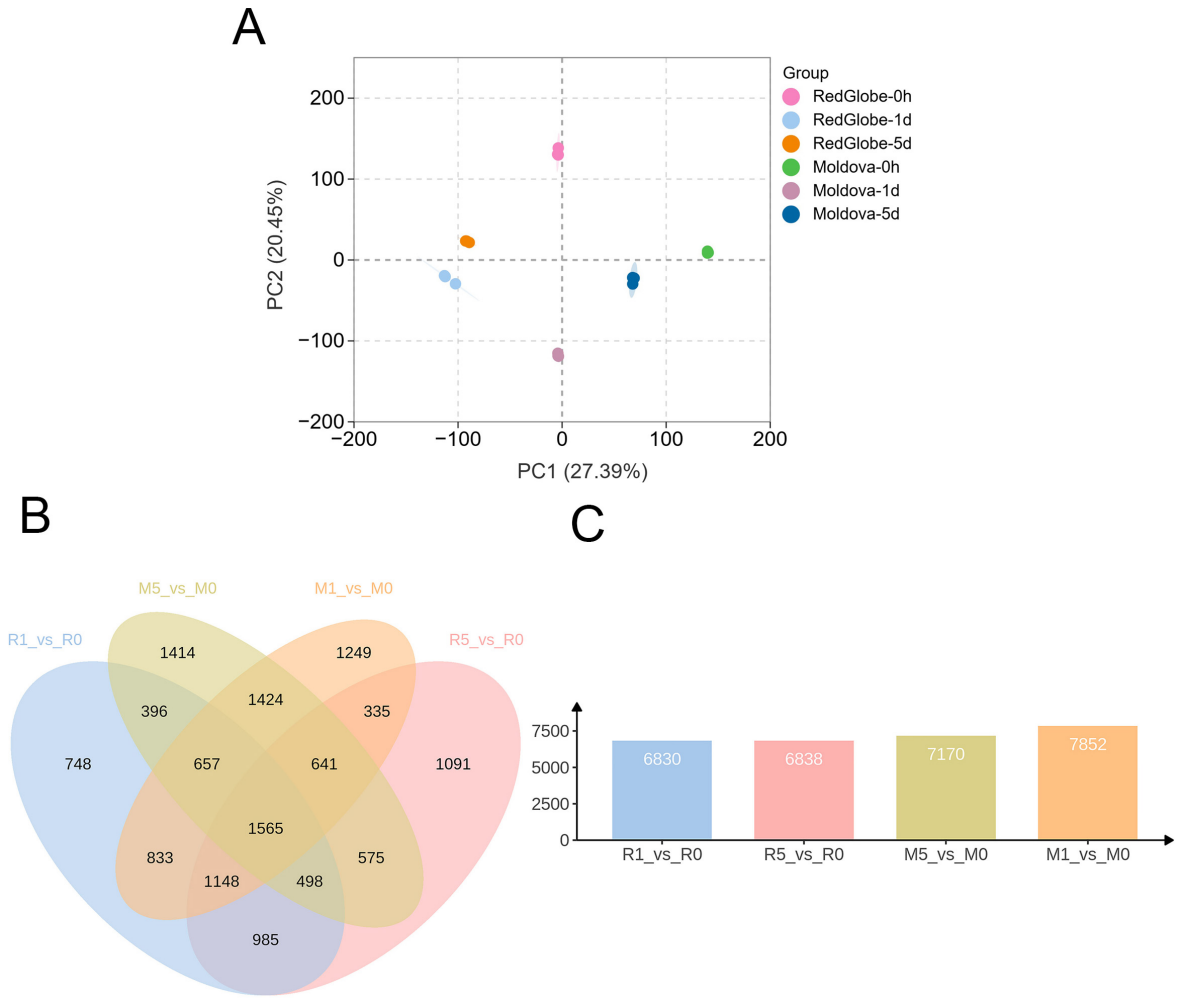
Overview of RNA-seq data. **(A)** Principal Component analysis (PCA) between samples. **(B)** Venn Diagram combined bar chart, showing the number of DEGs in each control group. **(C)** The number of differentially expressed genes in the four groups: M1_vs_M0, M5_vs_M0, R1_vs_R0, R5_vs_R0 (M0: Moldova-0h, M1: Moldova-1d, M5: Moldova-5d, R0: RedGlobe-0h, R1: RedGlobe-1d, R5: RedGlobe-5d).

**Figure 5:**
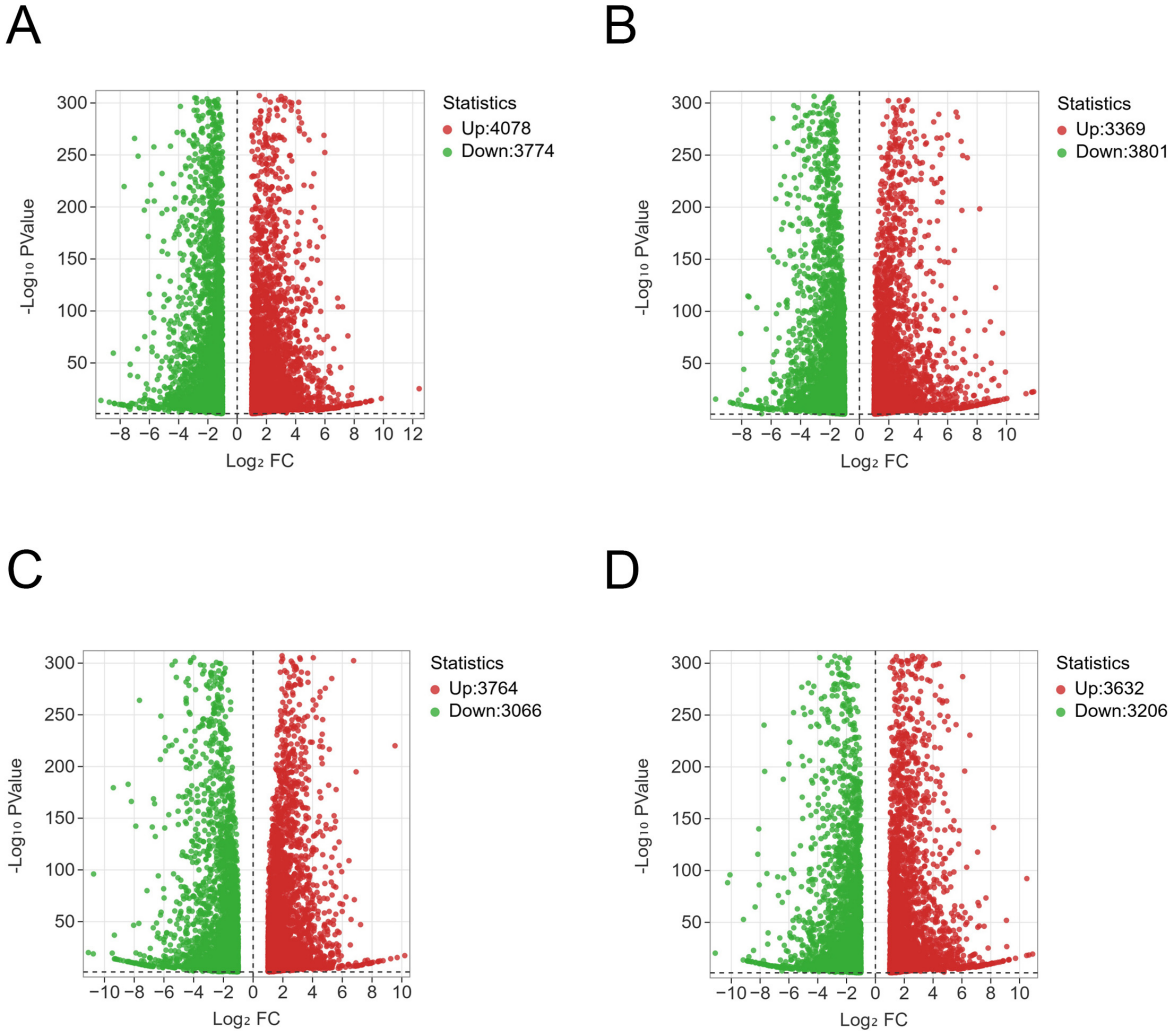
Volcano plots for differentially expressed genes (DEGs) M1_vs._M0, M5_vs_M0, R1_vs_R0, R5_vs_R0. The log_2_fold change (X-axis) is plotted against the log_10_FDR (Y-axis). Red dots and green dots represent DEGs that were either up-regulated or down-regulated. Gray dots denote genes that were not significantly different between treatments and mock. **(A)** M1_vs_M0. **(B)** M5_vs_M0. **(C)** R1_vs_R0. **(D)** R5_vs_R0 (M1_vs_M0, M5_vs_M0, R1_vs_R0, R5_vs_R0 (M0: Moldova-0h, M1: Moldova-1d, M5:Moldova-5d, R0: RedGlobe-0h, R1: RedGlobe-1d, R5: RedGlobe-5d).

Next-generation RNA sequencing (RNA-Seq) has been widely used to investigate global transcriptional changes in response to biotic stress. To investigate the differences in gene expression profiles between the two grape cultivars in response to *P. viticola* infection, transcriptome sequencing was performed on leaf samples of Moldova and Red Globe at three time points: 0 h, 1 dpi, and 5 dpi after pathogen invasion. Quality assessment revealed that the clean data yield for each sample exceeded 6 Gb, with the Q30 base percentage ≥92% (Table 1). These results confirmed the generation of high-quality RNA-seq data suitable for subsequent bioinformatic analyses. Principal component analysis (PCA) demonstrated distinct separation between the two grape cultivars at different post-inoculation time points. Three biological replicates of each cultivar at the same time point showed clustered PCA values, indicating the excellent sample reproducibility (Figure 4A). There was a total of 1,565 common differentially expressed genes (DEGs) identified across all four comparison groups (M1_vs_M0, M5_vs_M0, R1_vs_R0, R5_vs_R0) based on Venn diagram analysis (Figure 4B) (M0: Moldova-0h, M1: Moldova-1d, M5: Moldova-5d, R0: RedGlobe-0h, R1: RedGlobe-1d, R5: RedGlobe-5d). Further, 748, 1,091, 1,249, and 1,414 uniquely expressed DEGs were detected in each of these four groups, respectively (Figure 4C). Furthermore, to make the visualization more intuitive, we have drawn a volcanic map to show the distribution of DEGs across the different treatments (Figures 5A–D). Detailed DEG statistics for each comparison group were as follows: the M1_vs_M0 group contained 7,852 DEGs (4,078 upregulated and 3,774 downregulated genes) (Figure 5A); the M5_vs_M0 group had 7,170 DEGs (3,369 upregulated and 3,801 downregulated) (Figure 5B); the R1_vs_R0 group harbored 6,838 DEGs (3,764 upregulated and 3,066 downregulated) (Figure 5C); and the R5_vs_R0 group included 6,830 DEGs (3,632 upregulated and 3,206 downregulated) (Figure 5D). In more detail, all of the related genes are listed in Supplementary Table S3.

**Table 1:** Data-mapped quality of the transcriptome.

**Sample**	**Raw reads**	**Clean reads**	**Clean base (G)**	**Error rate (%)**	**Q20 (%)**	**Q30 (%)**	**GC content (%)**
Moldova-0h-1	49,817,292	48,122,598	7.22	0.03	97.65	93.42	45.47
Moldova-0h-2	46,955,516	45,386,204	6.81	0.03	97.63	93.27	45.53
Moldova-0h-3	47,892,940	45,980,262	6.90	0.03	97.62	93.23	45.52
Moldova-1d-1	51,572,684	49,353,298	7.40	0.03	97.55	93.15	45.93
Moldova-1d-2	49,709,862	47,583,462	7.14	0.03	97.61	93.31	45.91
Moldova-1d-3	47,872,700	45,757,680	6.86	0.03	97.34	92.73	45.86
Moldova-5d-1	49,046,376	46,541,788	6.98	0.03	97.72	93.58	45.85
Moldova-5d-2	46,707,878	44,667,012	6.70	0.03	97.67	93.39	45.97
Moldova-5d-3	49,778,932	47,504,010	7.13	0.03	97.7	93.42	46.13
RedGlobe-0h-1	42,571,066	40,591,264	6.09	0.03	97.59	93.18	45.01
RedGlobe-0h-2	54,761,722	52,033,994	7.81	0.03	97.22	92.42	45.05
RedGlobe-0h-3	47,143,920	44,961,042	6.74	0.03	97.22	92.41	45.11
RedGlobe-1d-1	52,775,244	50,149,410	7.52	0.02	98.38	95.02	45.64
RedGlobe-1d-2	52,146,754	49,692,666	7.45	0.03	97.65	93.37	45.94
RedGlobe-1d-3	52,218,330	49,538,396	7.43	0.03	97.65	93.37	45.52
RedGlobe-5d-1	46,403,110	44,522,952	6.68	0.03	97.67	93.43	46.22
RedGlobe-5d-2	52,508,630	50,215,688	7.53	0.03	97.47	93.03	46.23
RedGlobe-5d-3	46,072,232	44,163,216	6.62	0.03	97.31	92.58	46.12

### Validation with qRT-PCR of RNA sequencing results

**Figure 6:**
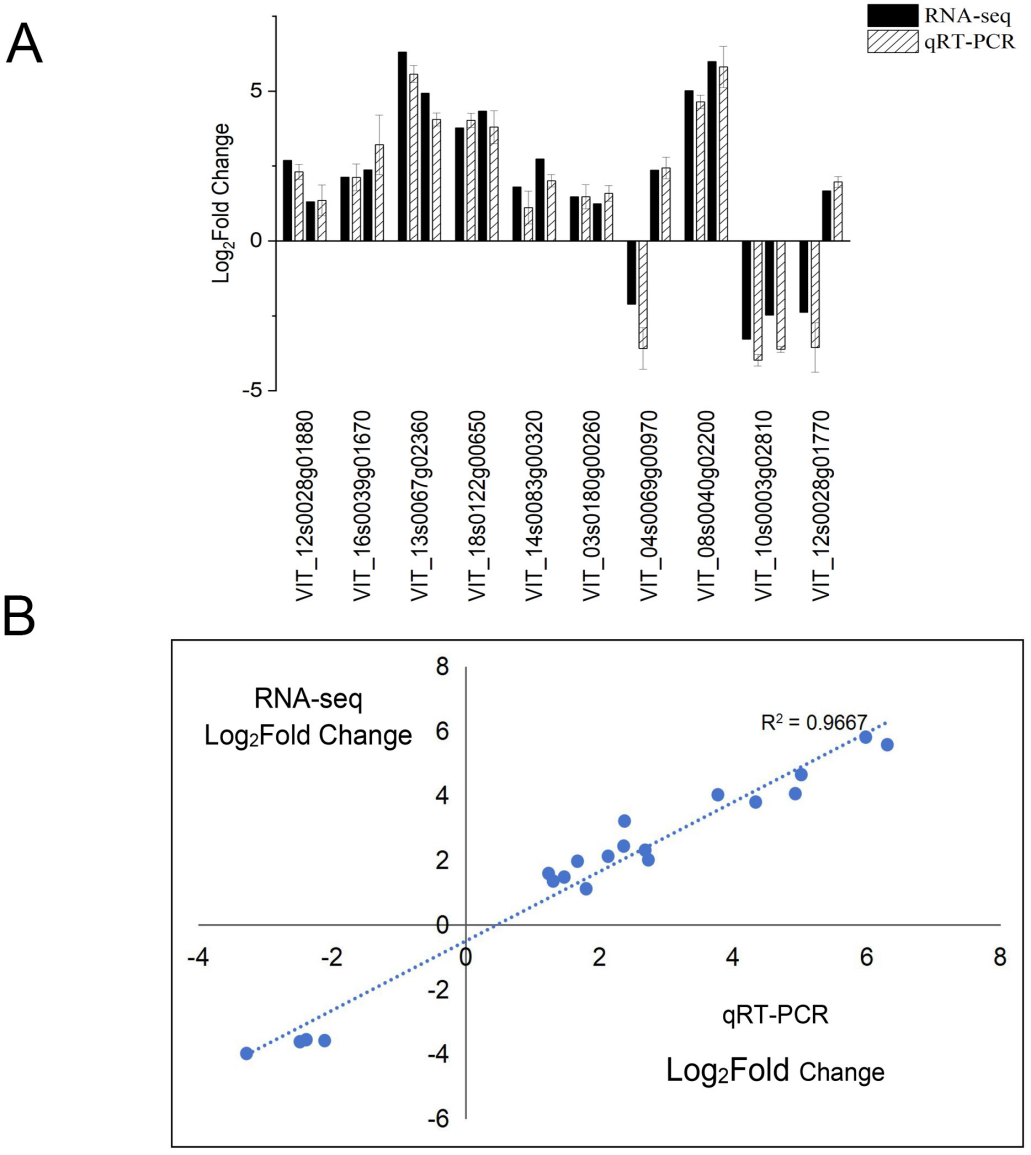
RT-PCR verification of RNA-seq results. **(A)** RT-qPCR and RNA-seq comparison between 10 randomly selected differentially expressed genes (DEGs) (VIT_03s0180g00260, VIT_12s0028g01770, VIT_16s0039g01670, VIT_10s0003g02810, VIT_04s0069g00970, VIT_12s0028g01880, VIT_08s0040g02200, VIT_13s0067g02360, VIT_14s0083g00320, VIT_18s0122g00650). Shown are the mean and SEM of the three independent biological replicates. **(B)** Regression analysis of gene expression ratios compared by RT-qPCR and RNA-seq analysis.

To verify the reliability of the RNA-seq data, a total of 10 genes were randomly chosen from two cultivars for qRT-PCR validation. The selected genes were as follows: for Moldova, VIT_12s0028g01880 (encoding resveratrol O-methyltransferase involved in stilbene metabolism, *ROMT*), VIT_16s0039g01670 (encoding a cinnamoyl-CoA reductase-like protein involved in lignin biosynthesis, *SNL6*), VIT_13s0067g02360 (encoding a class III peroxidase, *POD4*), VIT_18s0122g00650, and VIT_14s0083g00320; as for Red Globe, VIT_03s0180g00260 (encoding cinnamyl alcohol dehydrogenase in lignin synthesis, *CAD1*), VIT_04s0069g00970, VIT_08s0040g02200 (encoding a peroxidase involved in stress responses, *POD21*), VIT_10s0003g02810, and VIT_12s0028g01770. The results showed a strong linear correlation between the fold-change values determined by RNA-seq and qRT-PCR (*R*^2^ > 0.9667) (Figures 6A, B), indicating that the transcriptome data are reliable for further bioinformatic and functional analyses in this study.

### Functional annotation and classification of DEGs

**Figure 7:**
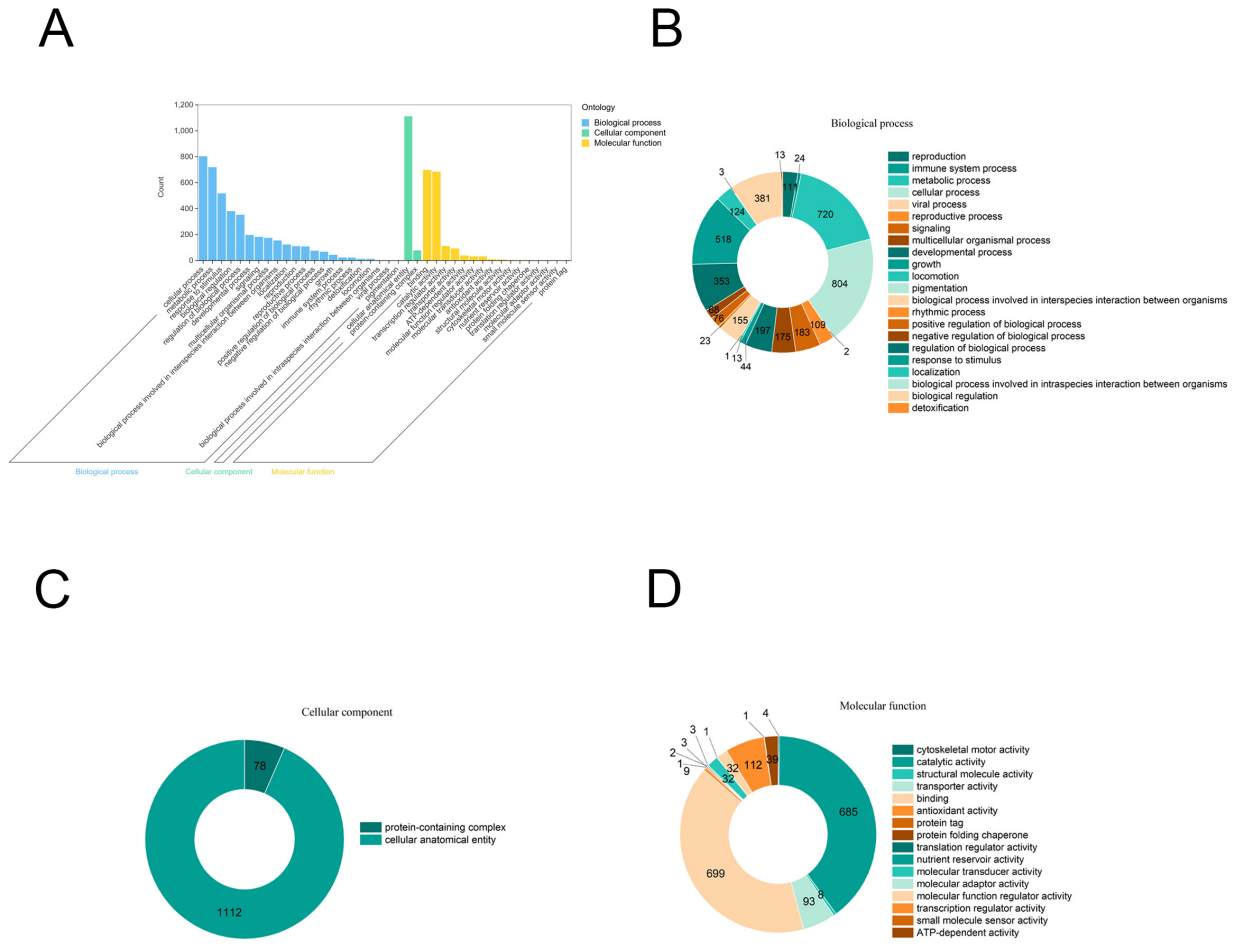
Gene Ontology (GO) annotations for differentially expressed genes (DEGs) in *Plasmopara viticola* inoculated grape leaves. **(A)** GO classifications of differential genes in *Plasmopara viticola* vs. control treatment with Moldova and RedGlobe. Pie charts showing the breakdown and prevalence of enriched GO terms in the following categories: biological process **(B)**, cellular component **(C)**, and molecular function **(D)**.

Gene Ontology (GO) is a system of standardized scientific classification used to annotate gene function systematically. To decipher potential biological processes and genes regulated under these treatments. We performed GO analysis (Figures 7A–D). A total of 1,565 DEGs were significantly enriched into 22, 2, and 16 secondary GO terms under the three main categories of biological process, cellular component, and molecular function, respectively (Figure 7A). Notably, these DEGs were highly enriched in six key secondary terms: metabolic process (GO: 0008152), cellular process (GO: 0009987), response to stimulus (GO: 0050896), cellular anatomical entity (GO: 0110165), catalytic activity (GO: 0003824), and binding (GO: 0005488) (Figure 7A). These enrichment patterns clearly reveal the multi-layered coordination mechanism of plant defense responses. The significant enrichment of “response to stimulus” and “binding” functions collectively points to early defense signaling events, including hormone perception and signal transduction, consistent with previous reports that multiple plant hormones play a central regulatory role (Pieterse et al., 2012). Moreover, the high enrichment of “metabolic process” and “catalytic activity” indicates that plants initiate extensive metabolic reprogramming in response to pathogen stress, particularly the biosynthesis of defense-related secondary metabolites. Therefore, GO analysis not only demonstrates that plant cells launch large-scale immune reprogramming but also suggests that key secondary metabolic pathways, such as hormone signaling, phenylpropanoid metabolism, and flavonoid synthesis, may represent the core functional modules of this process.

To gain deeper insights into the systematic biological functions of DEGs in response to *P. viticola* infection, Kyoto Encyclopedia of Genes and Genomes (KEGG) pathway enrichment analysis was carried out on the identified DEGs. Based on data analysis, a total of 1,359 DEGs were mapped to 129 KEGG pathways. All pathways were ranked in descending order based on the number of annotated DEGs, and 10 significantly enriched pathways (*P* ≤ 0.05) were selected for subsequent analysis (Table 2). Among the significantly enriched pathways, the plant hormone signal transduction pathway, phenylpropanoid biosynthesis pathway, and flavonoid biosynthesis pathway are particularly prominent. These three pathways not only rank highly in terms of enrichment significance and the number of included DEGs, but their biological functions also collectively form a coherent defense chain spanning from pathogen recognition and signal transduction (hormone signaling), to the synthesis of key defense metabolite frameworks (phenylpropanoid metabolism), and finally to the production of specific antimicrobial compounds (flavonoid synthesis).

**Table 2:** The Kyoto encyclopedia of genes and genomes (KEGG) pathways (top 10) enriched by differentially expressed genes (DEGs) under treatments.

**KEGG pathway ID**	**Description**	**Gene number**	***P*-value**
ko04075	Plant hormone signal transduction	62	0.003029288
ko00940	Phenylpropanoid biosynthesis	27	0.002796805
ko00941	Flavonoid biosynthesis	24	0.001691762
ko00945	Stilbenoid, diarylheptanoid, and gingerol biosynthesis	16	0.000536046
ko00360	Phenylalanine metabolism	12	0.005497712
ko00960	Tropane, piperidine, and pyridine alkaloid biosynthesis	10	0.006990555
ko00905	Brassinosteroid biosynthesis	10	0.008469491
ko00260	Glycine, serine, and threonine metabolism	10	0.032408128
ko00073	Cutin, suberine, and wax biosynthesis	9	0.01267225
ko00196	Photosynthesis-antenna proteins	4	0.019915522

### Differentially expressed genes involved in plant hormone signal transduction pathway

Plant hormones act as critical regulators in activating specific defense signaling pathways in response to pathogen challenge. To investigate the response of the plant hormone signal transduction pathway (ko04075) in resistant Moldova and susceptible Red Globe grape cultivars during *P. viticola* infection. A total of 62 DEGs were identified in this pathway (Supplementary Tables S4-1, S4-2), which involved Salicylic Acid (SA), Jasmonic acid (JA), Ethylene (ET), Abscisic acid (ABA), and Auxin signaling pathways. SA plays a key role in resistance to biotrophic pathogens; it has been reported that SA enhances the resistance of grape leaves to *P. viticola* by inducing the expression of PR (disease-related) genes (Rahman et al., 2022). In our study, the pathogenesis-related protein 1 gene (*VvPR1*, VIT_03s0097g00700) was significantly upregulated at 5 dpi in Moldova, whereas it was continuously downregulated in Red Globe, indicating that the resistant cultivar has an active SA-dependent signaling pathway (Supplementary Table S4-2). In our study, the JA-related jasmonic acid-amide synthase *VvJAR1* (VIT_12s0059g01870) showed upregulated expression characteristics in the leaves of Moldova and Red Globe after 1 dpi and 5 dpi of *P. viticola* inoculation (Supplementary Tables S4-1, S4-2), and the expression trends were consistent in both types of grapes. The protein regulatory factor TIFY family members (e.g., VIT_01s0146g00480, VIT_10s0003g03790) were downregulated in both grape samples at 1 dpi and 5 dpi, with VIT_10s0003g03790 showing a more significant downregulation in the Red Globe leaves at 5 days (Supplementary Table S4-2). The ethylene response transcription factor VIT_05s0049g0051 was upregulated in the Moldova leaves at 1 dpi and 5 dpi, but downregulated in Red Globe leaves. The abscisic acid (ABA) signal sensor gene *VvSUC11* (VIT_05s0077g01550) and the auxin receptor gene *VvTIR1* (VIT_07s0104g01320) were continuously upregulated in Moldova (Supplementary Tables S4-1, S4-2), suggesting that ABA and auxin signaling pathways may act synergistically to enhance the stress response during *P. viticola* infection. Notably, *VvWAK5* (VIT_10s0003g05130), which encodes a wall-associated receptor kinase involved in cell wall perception and reinforcement, was dramatically upregulated in Moldova, with a much higher upregulation amplitude than in the susceptible cultivar. The differential expression of these genes indicates that plants activate their defense responses against *P. viticola*, involving multiple hormone signaling pathways.

### Differentially expressed genes related to phenylpropane metabolism

The phenylpropanoid metabolic pathway is a core secondary metabolic pathway in plants, responsible for the biosynthesis of lignin, flavonoids, phenolic compounds, and other disease resistance-associated metabolites, and it also plays a pivotal role in mediating plant defense responses against pathogen invasion (Vogt, 2010; Dixon, 1995). In this study, transcriptome analysis identified 27 DEGs in the phenylpropanoid metabolic pathway between the resistant Moldova and susceptible Red Globe at different post-inoculation time points (Supplementary Tables S4-3, S4-4).

In the resistant Moldova cultivar, multiple DEGs in the phenylpropanoid metabolic pathway were significantly upregulated as early as 1 dpi. For instance, VIT_03s0017g01490 (encoding a stilbene synthase-like protein) was upregulated; VIT_03s0180g00260, encoding cinnamyl alcohol dehydrogenase (*CAD*), a key enzyme in lignin biosynthesis, was upregulated (Supplementary Table S4-3). These results suggest that key lignin synthesis enzymes are strongly induced in the early stage of infection. VIT_07s0031g00350, encoding caffeoyl-CoA O-methyltransferase (*VvCCoAOMT*), which promotes the methylation of lignin monomers, was upregulated (Supplementary Tables S4-3, S4-4). The rapid upregulation of these genes may facilitate cell wall reinforcement and lignification, thereby inhibiting pathogen invasion. Additionally, some peroxidase-encoding genes (VIT_07s0191g00050, VIT_08s0040g02200, VIT_08s0058g00990) were significantly upregulated in Moldova at 1 dpi; among them, VIT_08s0040g02200 showed an extremely high upregulation fold change of 6.35-fold (Supplementary Table S4-3). These peroxidases are involved in lignin polymerization and ROS burst, which may collectively enhance the resistance of the cell wall to pathogen infection.

### Differentially expressed genes involved in flavonoid biosynthesis pathway

Next, the DEGs involved in the flavonoid biosynthesis pathway under pathogen infection were analyzed. A total of 24 DEGs were identified in this pathway (Supplementary Tables S4-5, S4-6). The analysis showed that the flavonoid biosynthesis pathway was rapidly activated in Moldova following pathogen infection, whereas the response in Red Globe was relatively slow. Chalcone synthase (*VvCHS*, VIT_14s0068g00930), as the first rate-limiting enzyme in flavonoid skeleton synthesis, was upregulated by 4.2-fold at 1 dpi and further dramatically upregulated by 12.9-fold at 5 dpi in Moldova (Supplementary Table S4-6), showing a sustained enhancement trend. Conversely, in Red Globe, it was only upregulated by 3.6-fold at 1 dpi and then downregulated by 2.7-fold at 5 dpi (Supplementary Table S4-6). This indicates that resistant varieties can continuously activate and enhance the synthesis of newly formed flavonoids. Flavanone 3-hydroxylase (*VvF3H*, VIT_18s0001g03470), a key node enzyme in the flavonol and anthocyanin synthesis branches, was extremely strongly and persistently induced in Moldova, with upregulation folds of 4.3-fold at 1 dpi and 9.7-fold at 5 dpi (Supplementary Table S4-6). However, in Red Globe, it was only upregulated 3.0-fold at 1 dpi and downregulated by 1.0-fold at 5 dpi (Supplementary Tables S4-5, S4-6). The repression at this key node may block the synthesis of downstream antibacterial flavonoids in the susceptible cultivar. Dihydroflavonol 4-reductase (*VvDFR*, VIT_15s0048g01000), a key branch-point enzyme for the synthesis of anthocyanins and proanthocyanidins (highly antibacterial metabolites), was specifically strongly activated at the late infection stage (5 dpi) in Moldova, with an upregulation fold of 4.19-fold. In Red Globe, it was repressed at 1 dpi (downregulated by 1.7-fold) and upregulated by 2.96-fold at 5 dpi (Supplementary Table S4-6), showing delayed induction and a much lower upregulation amplitude than the resistant cultivar.

UDP-glucosyltransferase (*VvUFGT*), which is responsible for flavonoid glycosylation, affects the stability and activity of end products. Its family members (e.g., VIT_12s0034g00080) showed sustained upregulation in Moldova (upregulated by 1.3-fold at 1 dpi and 3.56-fold at 5 dpi), whereas the upregulation amplitude was small and insignificant in Red Globe (Supplementary Tables S4-5, S4-6). From a global perspective, the entire gene network of Moldova, from initial flavonoid skeleton synthesis (VIT_14s0068g00930) and key modification (VIT_04s0023g03370) to end-product orientation (VIT_15s0048g01000), was synergistically continuously activated. Conversely, Red Globe exhibited slow initiation, weak response intensity, and late-stage disorder in pathway activation, especially the severe repression of the key gene VIT_04s0023g03370 at the late infection stage (Supplementary Table S4-6), suggesting the interruption of its defense metabolic flux. Together, the expression of these genes involved in the flavonoid biosynthesis pathway varied across different cultivars.

### Expression of DEGs involved in stilbenoid, diarylheptanoid, and gingerol biosynthesis pathway

The stilbenoid biosynthesis pathway (ko00945) is an important, specific disease-resistant secondary metabolic pathway in grapes and other plants. By comparing the dynamic gene expression of resistant Moldova and susceptible Red Globe at different times, a total of 16 DEGs were identified in this pathway (Supplementary Tables S4-7, S4-8). In our results, stilbene synthase (*STS*), the key rate-limiting enzyme for stilbenoid synthesis that catalyzes resveratrol skeleton formation, and the gene VIT_16s0100g01100 were specifically strongly induced in Moldova, upregulated by 3.25-fold at 1 dpi and further dramatically upregulated 4.38-fold at 5 dpi (Supplementary Tables S4-7, S4-8). Conversely, VIT_16s0100g01100 was even repressed at 1 dpi (downregulated by 3.5-fold) in Red Globe, but was upregulated by 2.17-fold at 5 dpi (Supplementary Tables S4-7, S4-8). This probably indicates that the resistant cultivar can precisely mobilize specific *STS* isomers for efficient synthesis and may optimize metabolic resource allocation by repressing other isomers. Resveratrol O-methyltransferase (*ROMT*, VIT_12s0028g01880), responsible for methylating resveratrol into more antibacterial derivatives (e.g., pterostilbene): the gene was induced in both cultivars, but the induction in Moldova was earlier and more efficient (up-regulated by 2.69-fold at 1 dpi and 1.315-fold at 5 dpi) (Supplementary Tables S4-7, S4-8). In Red Globe, strong induction occurred at the late stage (up-regulated by 3.62-fold at 5 dpi) (Supplementary Table S4-8). However, in the pathway analysis, the expression of many Stilbene synthase (*STS1, STS3, STS4, STS5*) was inhibited to varying degrees after inoculation with *P. viticola* (Supplementary Tables S4-7, S4-8). The analysis revealed a distinct characteristic: the regulation of stilbenoid synthesis genes in Moldova showed high precision and dynamics rather than simple global upregulation, specifically manifested as strong specific induction of core synthesis genes, whereas the response in Red Globe was relatively slow and lacked effective activation of core synthesis genes.

## Discussion

The increasing severity of grape downy mildew disease has caused significant losses in global grape production. It has been reported that the infection of the grape *P.viticola* leads to oxidative damage of plant cells, which subsequently activates the phenylpropanoid metabolic pathway, synthesizing phenylpropanoid secondary metabolites and key antioxidant enzymes to enhance the plant's disease resistance (Arshad et al., 2018; Yasmin et al., 2023), and existing studies have confirmed that pathogen infection promotes the biosynthesis of phenylpropanoid metabolites such as lignin, flavonoids, and total phenols, thereby enhancing plant disease resistance (Qu et al., 2022; Li N. et al., 2024). In this study, the resistant cultivar Moldova exhibited markedly higher accumulations of flavonoids, chlorogenic acid, total phenols and lignin in leaves at 1 dpi with *P. viticola* compared with the susceptible Red Globe, which demonstrates that these phenylpropanoid metabolites are core defensive components mediating the early resistance response to *P. viticola* infection (Figure 2), indicating that these phenylpropanoid metabolites play an important role in resisting pathogen infection. Notably, “phenylpropanoid biosynthesis” was identified as the core enriched pathway mediating resistance to *P. viticola* (Supplementary Tables S4-3, S4-4), and its metabolites are indispensable for regulating disease- resistance metabolism, especially playing a prominent role in disease-resistant varieties. Lignin is an important branch of phenylpropanoid metabolism. Studies have shown that transgenic sugarcane with overexpressed *ScDIR* gene has enhanced resistance to sugarcane smut (Sporisorium scitamineum) (the *DIR* is involved in the synthesis of the lignin precursor farnesol) (Li X. et al., 2024), when *Rhizoctonia solani* Kühn infects and causes potato black spot disease, the lignin synthesis pathway is significantly activated, and its content continuously increases throughout the infection process, and is positively correlated with the contents of total phenols, flavonoids, etc., playing a key role in potato's resistance to black spot disease (Yang et al., 2024). These studies all confirm that lignin biosynthesis can effectively enhance plant disease resistance. Consistent with these findings, lignin biosynthesis was specifically activated in Moldova to mediate resistance, with its accumulation significantly higher than that in Red Globe. This early and robust lignin induction is a typical defensive characteristic of resistant grape cultivars against *P. viticola* (Figure 2). Meanwhile, the differentially expressed genes of disease-resistant varieties are significantly enriched in the lignin biosynthesis branch of the phenylpropanoid pathway and are significantly upregulated during grape downy mildew infection (Supplementary Table S2). The key enzymes of the lignin biosynthesis pathway, cinnamoyl-CoA reductase (*CCR*) and *CAD*, show high transcriptional abundance and are significantly upregulated in disease-resistant varieties (Supplementary Table S2). In our analysis, the genes encoding the key enzyme for lignin biosynthesis, cinnamyl alcohol dehydrogenase (VIT_03s0180g00260) and the gene encoding caffeoyl-CoA O-methyltransferase (VIT_07s0031g00350) were significantly induced (Supplementary Tables S4-3, S4-4). Together with the specific induction of these key structural genes, the high lignin accumulation in Moldova collectively reveals that resistant varieties possess a more responsive lignin biosynthesis pathway under *P. viticola* stress, which enables rapid physical barrier construction at the infection site (Figure 2), these results jointly indicate that disease-resistant varieties have a stronger induction of the lignin biosynthesis pathway under *P. viticola* stress. For other metabolites, as it has been shown, 2-phenylethanol and β-cyclodextrin aldehyde can act as resistance inducers for grape downy mildew, and these volatile organic compounds reprogram the flavonoid, phenylpropanoid, and terpene metabolism, activating the phenylpropanoid metabolic pathway to resist the invasion of *P. viticola*, which is consistent with the previous findings (Avesani et al., 2025; Chandrasekaran and Chun, 2018; Boateng et al., 2024). These results collectively elucidate that phenylpropanoid secondary metabolites, particularly lignin, act as both chemical and physical defensive mediators essential for the grape resistance response to *P. viticola*.

The phenylpropanoid pathway is initiated by successive catalysis of phenylalanine ammonia-lyase (PAL), cinnamic acid-4-hydroxylase (C4H), and 4-caffeoyl-CoA ligase (4CL), which are crucial for the generation of various downstream metabolites such as lignin, flavonoids, anthocyanins, and other specific metabolites (Yao et al., 2021). This study further found that PPO and POD activities in resistant grape leaves were significantly elevated at 5 dpi in response to *P. viticola* relative to susceptible varieties; these enhanced antioxidant enzyme activities are tightly linked to the phenylpropanoid pathway, and jointly confer a stronger ROS scavenging capacity to resistant varieties under pathogen stress (Figure 3). This is also applicable to other crops, for example, after infection with tomato yellow leaf curl virus (TYLCV), the activities of superoxide dismutase (SOD), peroxidase (POD), and catalase (CAT) in the leaves significantly increased, providing important ideas for the selection of resistant varieties and the prevention and control of tomato diseases (Ni et al., 2025). This interconnection between enhanced antioxidant enzyme activity and phenylpropanoid metabolism further validates the pivotal and multifaceted role of the phenylpropanoid pathway in grape-specific resistance to *P. viticola*.

Consistent with previous research on grape-oomycete interactions (Chen et al., 2023; Cao et al., 2024), transcriptomic analysis identified several core pathways responsive to *P. viticola* infection, including plant-pathogen interaction, secondary metabolite biosynthesis, metabolic pathways, and plant hormone signal transduction; these pathways form a coordinated regulatory network that orchestrates the grape defense response to *P. viticola*. Plant hormones, as core regulatory factors of specific defense signaling pathways under pathogen stress, require precise regulation of their signaling networks, which is key for plants to initiate defense responses (Belhadj et al., 2006). We found that disease-resistant varieties can effectively activate SA-dependent defense signaling pathways and enhance inhibition of downy mildew by efficiently expressing *VvPR1* (Supplementary Table S4-1). The JA-amide synthase gene *VvJAR1* (VIT_12s0059g01870), mediated by JA signal activation, may be the basic defense response of grapes against downy mildew infection. Its catalytic action in combining JA with amino acids to form the active forms (such as JA-Ile) lays the foundation for subsequent defense signal transmission (Supplementary Tables S4-1, S4-2). The ethylene response transcription factor VIT_05s0049g0051 was continuously upregulated at both 1 dpi and 5 dpi in Moldova, whereas an opposite expression pattern was observed in Red Globe (Supplementary Tables S4-1, S4-2); this sustained ET pathway activation in resistant varieties constructs a critical regulatory bridge between hormone signaling and secondary metabolism, ensuring the persistent induction of phenylpropanoid and other defensive metabolites. Additionally, the abscisic acid signal sensor gene *VvSUC11* (VIT_05s0077g01550) and the auxin receptor gene *VvTIR1* (VIT_07s0104g01320) are continuously upregulated in Moldova, suggesting that the ABA and auxin signaling pathways may form a synergistic network to enhance the stress adaptation ability of plants to downy mildew infection, providing multi-dimensional regulatory support for grape resistance.

This study further revealed that *P. viticola* infection not only upregulated the lignin synthesis branch of the phenylpropanoid pathway but also specifically activated STS family genes (e.g., VIT_16s0100g01100 and VIT_12s0028g01880) (Supplementary Tables S4-7, S4-8), which reflects a strategic co-activation of two key defensive branches in the phenylpropanoid pathway under pathogen stress. The stilbenoid synthesis mediated by *STS* and lignin synthesis are two important branches downstream of phenylpropanoid metabolism, sharing a common precursor derived from phenylalanine (Liu et al., 2010). Under pathogen stress, plants may simultaneously enhance the synthesis of STS-dependent phytoalexins (such as resveratrol) and lignin polymerization through upstream universal transcriptional regulation. It has been reported that the accumulation of resveratrol can prevent motile spores from reaching the stomata and protect grape leaves, effectively reducing the risk of grape powdery mildew infection (Krzyzaniak et al., 2018). Resveratrol functions as a key antibacterial chemical defense compound, while lignin strengthens cell wall formation to provide a physical barrier, jointly constructing a comprehensive defense system (Wang, 2018). These findings comprehensively elucidate the unique regulatory strategy of grapevines in coordinating different branches of the phenylpropanoid pathway during resistance response to *P. viticola*, namely the simultaneous induction of physical defense (lignin) and chemical defense (stilbenes) to construct a comprehensive and layered defensive system.

In conclusion, integrated physiological and transcriptomic analyses revealed that the resistant cultivar Moldova confers a prominent defense advantage over the susceptible Red Globe after *P. viticola* infection, which is manifested in three interconnected defensive characteristics: (1) Sustained and high accumulation of phenylpropanoid secondary metabolites: flavonoids, chlorogenic acid and total phenols exert a strong chemical inhibitory effect on *P. viticola*, while lignin reinforces cell wall physical barrier function, and the chemical-physical dual defense mechanism jointly blocks pathogen invasion at the early infection stage; (2) Efficient and persistent antioxidant enzyme activity: the high PPO and POD activities rapidly scavenge ROS induced by pathogen infection, thus preventing the spread of oxidative damage and ensuring the normal operation of phenylpropanoid metabolic pathways; (3) Enhanced membrane system stability: lower MDA content in Moldova alleviates stress-induced membrane damage, which preserves the normal physiological and metabolic functions of grape cells and provides a structural basis for the implementation of defensive responses.

## Conclusion

In this study, we compared changes in phenylpropane metabolite contents and transcriptome gene expression differences between downy mildew-resistant and susceptible grape cultivars before and after *Plasmopara viticola* infection. The results showed that *Plasmopara viticola* infection induced the expression levels of DEGs related to lignin biosynthesis, stilbene synthase (*STS*) biosynthesis, and regulation in the phenylpropane metabolic pathway; notably, the gene expression levels in resistant materials were significantly higher than those in susceptible materials. These findings provide an important insight for screening core defense genes and breeding downy mildew-resistant grape cultivars.

## Data Availability

The RNA-seq data for the Moldova and Red Globe strains inoculated with the *P. viticola* at 0 h, 1 dpi, and 5 dpi are available at NCBI gene expression omnibus server (accession no: SAMN54461750).
