# Supplementary material for: Phenylpropanoid metabolites from grape leaves contribute to strong defense roles against downy mildew based on physiological and transcriptomic analyses
Source: Front Microbiol. 2026 Mar 17;17:1805591. doi: 10.3389/fmicb.2026.1805591 (PMC13035788; doi:10.3389/fmicb.2026.1805591)
Supplement: Supplementary file 1 [file Table_1.docx]

TABLE S1 Grading criteria for grape downy mildew on leaves.

| Resistance grade | Disease performance  (Lesion area proportion) | Disease index range | Resistance evaluation |
| --- | --- | --- | --- |
| Grade 0 | No lesions on the whole leaf | Disease index = 0 | Immune (IM) |
| Grade 1 | ＜5% | 0 ＜ DI ≤ 5 | Highly resistant (HR) |
| Grade 3 | 6%~25% | 5 ＜ DI ≤ 20 | Resistant (R) |
| Grade 5 | 26%~50% | 20 ＜ DI ≤ 40 | Moderately resistant (MR) |
| Grade 7 | 51%~75% | 40 ＜ DI ≤ 60 | Susceptible (S) |
| Grade 9 | ＞75% | 60 ＜ DI ≤ 100 | Highly susceptible (HS) |

TABLE S2 Primer sequence used in this study.

| Gene | Forward | Reverse | Product size |
| --- | --- | --- | --- |
| VIT_03s0180g00260 | GTTCGTGGTTGATGTCGCTG | AACAGCGCCGGGTTAATCAT | 85 |
| VIT_12s0028g01770 | ACAAGCTATGCACAAGCCAG | AGGAATTAGTTTGGGCCACTG | 197 |
| VIT_16s0039g01670 | TTCACATCCTCCATCACGGC | CTAGTGCCCATGCTGTCCTC | 160 |
| *VvActin1* | GATTCTGGTGATGGTGTGAGT | GACAATTTCCCGTTCAGCAGT | 168 |
| VIT_10s0003g02810 | TGCTGCAGGCTCTTTCTACC | TGGAGGGAACCACATCTTGC | 92 |
| VIT_04s0069g00970 | TTCATGCTCGCAGACTACGG | GAGTTGCAGTGTGGATCAGC | 86 |
| VIT_12s0028g01880 | CCGTCTCATGCGCATTCTTG | CACCAAGGGCCTTATGCTCA | 142 |
| VIT_08s0040g02200 | CCCGCTTTCAATCCATTGGC | ACTGCCTTTGGGTCAGGTTC | 190 |
| VIT_13s0067g02360 | TGGCCATTGCTGCTAGAGAC | AGCTAAGGCAACCAAGTCCC | 197 |
| VIT_14s0083g00320 | CGAGTCACCATTCAAAAAGAAGTGG | GGCAACCTCTGAAAGCATCAC | 139 |
| VIT_18s0122g00650 | CCTGCCTAGGATTCCCCTTG | AAAGGGCTAAATTGGGTTTGGA | 187 |
